# Imaging spectrum of horseshoe lung: A series of four cases

**DOI:** 10.4102/sajr.v28i1.2766

**Published:** 2024-04-12

**Authors:** Vikas Yadav, Rama Anand, Pooja Abbey, Samrin Haq

**Affiliations:** 1 Department of Radiodiagnosis, Lady Hardinge Medical College, New Delhi, India

**Keywords:** horseshoe lung, pseudo-horseshoe lung, scimitar syndrome, lung hypoplasia, congenital lung anomaly

## Abstract

Horseshoe lung (HL) is a rare congenital anomaly represented by the fusion of both lungs, posterior to the heart, and is typically associated with various bronchopulmonary and cardiovascular malformations. Multi-detector contrast enhanced CT is the imaging modality of choice to demonstrate the pathology and associated malformations. There has been inconsistency in the nomenclature used for such cases in literature. To resolve ambiguity, the authors emphasise that only two terms: HL and pseudo-HL be used on imaging to describe variants of this congenital malformation.

**Contribution:**

A description of the imaging features in four cases of HL, with their associated malformations and a review of the nomenclature.

## Introduction

Fusion of both lungs posterior to the heart is described as horseshoe lung (HL). It is a rare congenital anomaly and is commonly associated with other complicated lung and cardiovascular malformations. The most common anomaly associated with these lesions is unilateral lung hypoplasia.^1^ In cases with HL, an isthmus of the lung parenchyma, usually arising from the hypoplastic lung, is seen posterior to the heart (usually posterior to the left atrium), and it may or may not demonstrate a pleural fissure at its interface with the contralateral lung. A few cases of previously documented HL have been associated with right lung hypoplasia and scimitar syndrome.^1,2^ However, an association with left lung hypoplasia is uncommon with only 11 such cases reported in the literature.^3^

This report describes four cases of HL, highlighting some rare associations. Two cases were associated with left lung hypoplasia, three cases showed anomalous pulmonary veins, one case demonstrated bilateral scimitar syndrome variants, and one case constituted a pseudo-scimitar syndrome. An association of HL with bilateral intralobar pulmonary sequestration was also seen in one case.

To resolve ambiguity, the authors emphasise the use of only two terms to describe HL on imaging: true HL and pseudo-HL. These can be differentiated by the absence and presence of a pleural fissure between the isthmus and contralateral lung parenchyma in true HL and pseudo-HL respectively, as described previously by Tosun et al.^2^

## Case series

All the presented cases were in children, aged between 7 days and 5 years who underwent contrast enhanced computed tomography (CECT) imaging of the chest for suspected congenital bronchopulmonary anomalies, based on clinical and chest radiographic findings.

### Case 1

**FIGURE 1:**
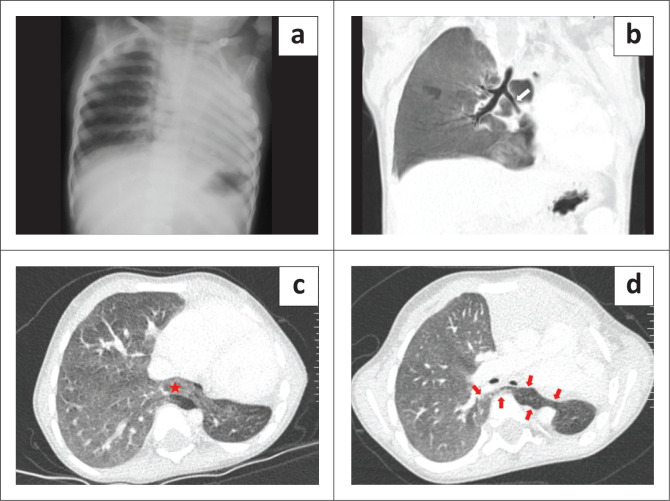
Horseshoe lung in a 2-year-old male associated with left lung hypoplasia. (a) Chest radiograph demonstrates a small, opaque left hemithorax with ipsilateral mediastinal shift and compensatory hyperinflation of the right lung. (b) Minimum intensity projection (MinIp) coronal image of the CT chest shows a hypoplastic left main stem bronchus (white arrow) with an absent left upper lobe branch. (c and d) Axial CT chest (lung window) demonstrates fusion of both lungs (red star) without any intervening fissure, with the isthmus of the midline lung parenchymal tissue (red arrows) extending up to the level of the tracheal bifurcation.

**FIGURE 2:**
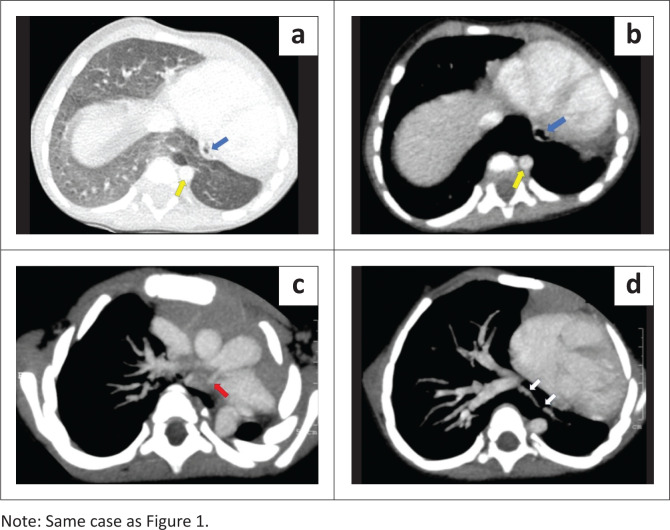
Contrast enhanced CT chest (a) lung window, (b) mediastinal window) images reveal that the fusion of the lung parenchyma lies posterior to the heart and the oesophagus (blue arrow) and anterior to the aorta (yellow arrow). Axial maximum intensity projection (MIP) images (c and d) demonstrate a hypoplastic left pulmonary artery at its origin (red arrow), and a single pulmonary vein (white arrows) draining the hypoplastic left lung, traversing through the isthmus of the true horseshoe lung and joining the right inferior pulmonary vein.

A 2-year-old male presented with complaints of poor weight gain and short stature. On imaging workup, the chest radiograph demonstrated an opaque left hemithorax with volume loss (Figure 1a). Contrast enhanced CT thorax revealed that the left lung was hypoplastic, comprising only the lower lobe. The left upper lobe bronchus was absent, suggesting left upper lobe agenesis (Figure 1b). Fusion of the posterobasal segments of the lower lobes of the right and left lungs was seen posterior to the heart (left and right atria). No isthmic fissure was visible, suggesting true HL (Figure 1c). Superiorly, the isthmus of the midline lung parenchymal tissue extended to the level of the tracheal bifurcation (Figure 1d). The isthmus was located posterior to the oesophagus and anterior to the aorta (Figure 2a, 2b). Bronchial supply to the isthmus was from a branch of the left bronchial artery. The left pulmonary artery was also hypoplastic, and coursed anterior and lateral to the hypoplastic left bronchus (Figure 2c). A single left pulmonary vein was seen traversing through the parenchymal isthmus of the HL and subsequently draining into the right inferior pulmonary vein (Figure 2d). Associated anomalies included duplication of the superior vena cava. The child had no respiratory complaints on initial presentation but was subsequently lost to follow-up.

### Case 2

A 7-day-old male presented with difficulty feeding and features of respiratory distress since birth. The neonate was tachypnoeic, with a respiratory rate of 64 breaths per minute, and required oxygen support. The chest radiograph (not shown) showed a small left hemithorax with ipsilateral mediastinal shift.

**FIGURE 3:**
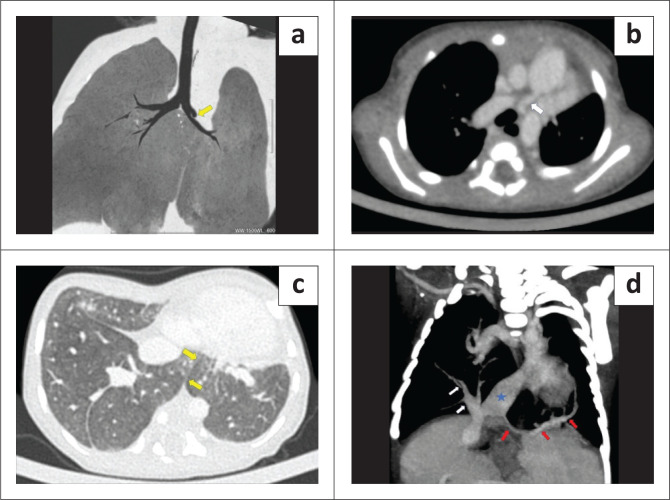
Pseudo-horseshoe lung in a 7-day-old male with left lung hypoplasia, total anomalous pulmonary venous return and multiple cardiovascular anomalies. (a) Coronal (minimum intensity projection [MinIP]) image of the contrast enhanced CT chest (lung window) demonstrates the rudimentary blind ending left upper lobe bronchus (arrow), absence of left upper lobe parenchyma and relatively narrow calibre of the left main bronchus in comparison with the right. (b) Axial maximum intensity projection (MIP) image shows the hypoplastic left pulmonary artery (arrow) arising from the main pulmonary artery. (c) Axial CT chest (lung window) demonstrates an isthmus of lung parenchyma joining the basal segments of both lungs posterior to the heart, with a thin intervening isthmic fissure (arrows). (d) Coronal oblique MIP image shows total anomalous pulmonary venous return with the right common anomalous pulmonary vein (white arrows) and the left single pulmonary vein (red arrows) both draining into the supra-hepatic inferior vena cava (star).

**FIGURE 4:**
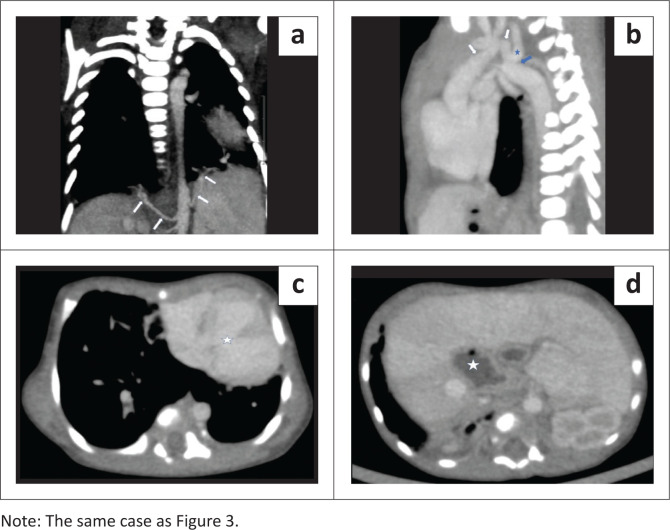
(a) Coronal oblique maximum intensity projection (MIP) image of the contrast enhanced computed tomography (CECT) chest demonstrates aberrant systemic arterial supply from the abdominal aorta to both lower lobes. (b) Sagittal oblique MIP image on mediastinal window shows a hypoplastic aortic arch (white arrows) with distinct narrowing of the aortic isthmus (blue arrow), just distal to the origin of the left subclavian artery (blue star), representing pre-ductal coarctation of the aorta. (c) Axial CECT image demonstrates a large ventricular septal defect (star). (d) Axial CT through the upper abdomen shows the midline position of the stomach (solid star), an absent spleen and a relatively large, transversely placed liver, suggesting situs ambiguous with right isomerism.

The CECT thorax revealed a hypoplastic left lung with a rudimentary blind ending left upper lobe bronchus and absence of left upper lobe parenchyma, suggesting left upper lobe aplasia (Figure 3a). The left pulmonary artery was also hypoplastic and showed an aberrant course anterolateral to the left main bronchus (Figure 3b). An isthmus of lung parenchyma was seen from the posterobasal segment of the right lower lobe, crossing the midline, and separated from the left lower lobe parenchyma by only a thin fissure, representing pseudo-HL morphology (Figure 3c). The left lower lobe bronchus was normal. Total anomalous pulmonary venous return was present in both lungs, suggesting bilateral scimitar syndrome variants. There was a single pulmonary vein draining the left lung, which continued as an anomalous vein, crossed the diaphragm, and drained into the inferior vena cava (IVC). The superior and inferior pulmonary veins on the right side also combined to form an anomalous common right pulmonary vein, which in turn drained into the supra-hepatic IVC (Figure 3d). Variant fissural anatomy was present in the right lung, with an absent oblique fissure and an incomplete accessory fissure containing the right anomalous common pulmonary vein within it. Aberrant systemic arteries arising from the abdominal aorta were present bilaterally, supplying the lower lobes (Figure 4a). The aortic arch was relatively narrow in calibre and a distinct narrowing of the aortic isthmus was present just distal to the origin of the left subclavian artery, representing pre-ductal coarctation of the aorta with aortic arch hypoplasia (Figure 4b). A large inlet ventricular septal defect was also seen (Figure 4c). Sections of the upper abdomen revealed a midline position of the stomach, absent spleen and a relatively large transversely placed liver, suggesting situs ambiguous with right isomerism (Figure 4d). The cardiovascular anomalies were also subsequently confirmed on echocardiography. Unfortunately, the neonate succumbed to the cardiac complications.

### Case 3

**FIGURE 5:**
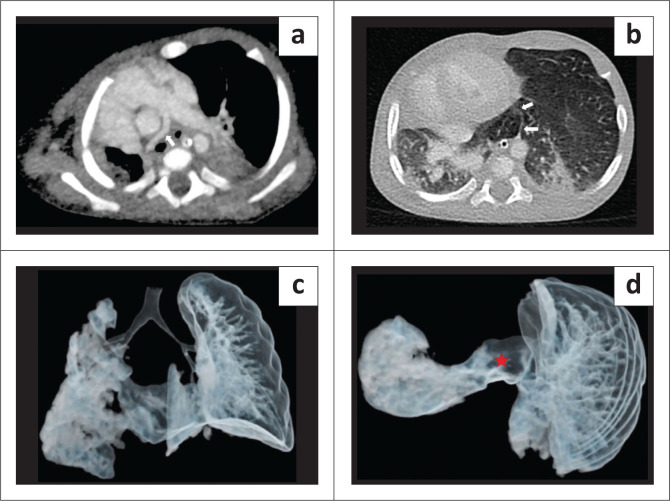
Pseudo-horseshoe lung in a 3-month-old male associated with right lung hypoplasia, anomalous unilateral single pulmonary vein and superimposed pulmonary infection. (a) Axial maximum intensity projection image of contrast enhanced CT chest (mediastinal window) shows the origin of the hypoplastic right pulmonary artery (arrow) from the main pulmonary artery. (b) Axial CT lung window image reveals fusion of the basal segments of the lower lobes with an intervening isthmic fissure (arrows). The isthmus of lung parenchyma is present posterior to the heart and anterior to the aorta and the oesophagus (as indicated by the nasogastric tube). An area of consolidation is present in the left lower lobe. (c and d) 3D volume rendered images demonstrate the fusion of the basal segments of both lungs via a parenchymal isthmus (red star) arising from the hypoplastic right lung.

**FIGURE 6:**
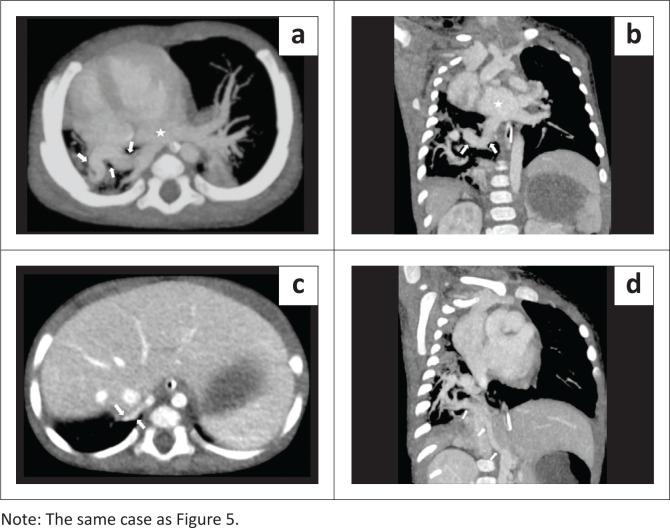
(a) Axial maximum intensity projection (MIP) image and (b) Coronal MIP image of the contrast enhanced computed tomography (CECT) chest reveal an anomalous unilateral single right pulmonary vein (arrows) that is tortuous and draining into the left atrium (white star). (c) Axial CT image and (d) oblique coronal MIP image of the CECT chest show an aberrant systemic artery (arrows) arising from the abdominal aorta and supplying the right basal lung parenchyma.

A 3-month-old male presented with features of severe pneumonia and raised total leucocyte counts (20 000/mm^3^). Chest radiograph (not shown) showed volume loss affecting the right hemithorax with ipsilateral shift of the mediastinum and inhomogeneous parenchymal opacities in both lungs. The CECT revealed hypoplasia of the right lung and right pulmonary artery (Figure 5a). Dextroposition of the heart was present with fusion of the basal segments of the right and left lower lobes in the midline, anterior to the aorta and the oesophagus. The isthmus of lung parenchyma originating from the right lower lobe formed a curvilinear fissure at the junction with the left lower lobe, representing a pseudo-HL morphology (Figure 5b–d). No fissure was present in the hypoplastic right lung. An anomalous unilateral single pulmonary vein was present on the right side, which was tortuous in course, but drained into the left atrium (Figure 6a, 6b). Areas of consolidation were present in both lungs, more on the right. A systemic artery arising from the abdominal aorta was also seen supplying part of the right lower lobe (Figure 6c, 6d). Blood culture revealed Klebsiella sepsis. The child received intravenous antibiotics and respiratory support demonstrating clinical improvement after treatment. No recurrent chest infections were noted on follow-up over the next 6 months.

### Case 4

**FIGURE 7:**
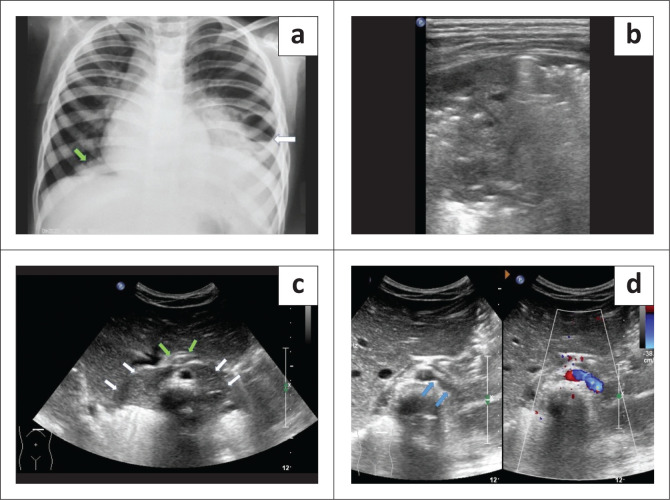
Bilateral intralobar pulmonary sequestration associated with true horseshoe lung in a 5-year-old girl with recurrent chest infections. (a) Frontal chest radiograph reveals consolidation with cavitation (white arrow) in the left mid and lower zones and patchy consolidation in the right lower zone, near the cardiophrenic angle (green arrow). (b) High frequency ultrasound image of the chest depicts the left lung consolidation. (c) Ultrasound image, transverse view through the epigastrium, shows abnormal soft tissue just above the diaphragm at the bases of both lower lobes (white arrows). This soft tissue is seen in continuity across the midline anterior to the aorta and posterior to the oesophagus (green arrows). (d) Transverse ultrasound image with colour doppler demonstrates an aberrant arterial branch (blue arrows) arising from the abdominal aorta and supplying the soft tissue, suggesting pulmonary sequestration.

A 5-year-old female presented with cough, respiratory difficulty and high-grade fever with chills for 3 days. She had a raised total leucocyte count (19 000/mm^3^). The patient had a significant past history of multiple episodes of lower respiratory tract infection since infancy. The chest radiograph showed consolidation with cavitation in the left mid and lower zones, along with a small patchy opacity near the right cardiophrenic angle (Figure 7a). Ultrasound of the chest showed areas of consolidation in the left infra-scapular and infra-axillary regions with a few small fluid-filled cavities within the consolidated lung (Figure 7b). Using a subcostal approach, abnormal hypoechoic soft tissue was seen on ultrasound just above the diaphragm, adjacent to the vertebrae, in the region of lung bases. This soft tissue was seen in continuity across the midline anterior to the aorta and posterior to the oesophagus (Figure 7c). A branch of abdominal aorta was seen supplying the soft tissue, which was suggestive of pulmonary sequestration (Figure 7d).

**FIGURE 8:**
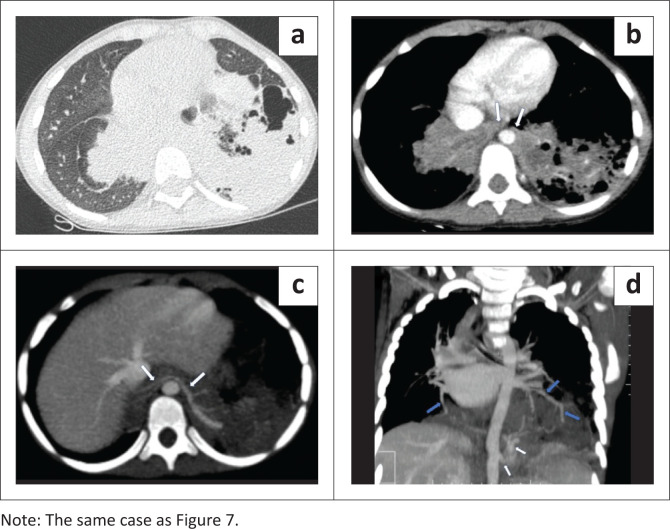
(a and b) Axial CT images (a, lung window and b, mediastinal window) confirm the presence of lung sequestration within the basal segments of both lower lobes, which are fused together in the midline anterior to the aorta (arrows). A partial accessory fissure is seen in the right lower lobe and consolidation with cavitation in the left lower lobe. (c) Axial maximum intensity projection (MIP) image shows an aberrant systemic arterial branch traversing through the bridging isthmus from left to right (arrows) and supplying the sequestered lung segments bilaterally. (d) Coronal MIP image demonstrates the origin of the systemic arterial branch (white arrows) from the abdominal aorta, as well as the area of consolidation in the left lower lobe. Bilateral inferior pulmonary veins (blue arrows) are draining the respective sequestered areas in each lung.

The CECT chest and upper abdomen confirmed the presence of bilateral intralobar lower lobe pulmonary sequestration, with arterial supply from a branch of the descending thoracic aorta at the T11 level (Figure 8 a–c). The branches of this systemic artery were seen to traverse through the midline isthmus from the left to the right (Figure 8c). Venous drainage of the pulmonary sequestration was into the inferior pulmonary veins on either side (Figure 8d). The CT showed consolidation in the left lower lobe, representing superimposed infection. The child showed marked clinical improvement after 2 weeks of intravenous antibiotics and interval surgery was recommended.

## Discussion

Spencer coined the term HL in 1962 for a congenital malformation in which the bases of both lungs are joined together across the midline by an isthmus of lung parenchyma.^4^ Multi-detector CECT is now the imaging modality of choice to diagnose HL, and has replaced conventional pulmonary angiography and bronchography, which were used traditionally.^5^

The primary feature of HL is the fusion of the right and the left lung through a defect in the parietal pleura, that is, the lung tissue is ensheathed within one common parietal pleural cavity. As a result, the right and left lung parenchyma may either be in direct continuity or may be separated by an isthmic fissure, which contains only two layers of visceral pleura, analogous to the fissures in a normal lung.^6^

Hawass et al. summarised the autopsy or operative findings of previous reported cases of HL, including cases showing an intervening isthmic fissure.^6^ Clements et al. described this displaced pulmonary segment crossing the midline as the ‘crossover’ segment.^7^ Figa et al. classified HL based on the pleural anatomy and proposed three patterns: (1) complete fusion of the parenchymal isthmus without any intervening pleura, (2) a pleural fissure along one side of the parenchymal isthmus, (3) a pleural fissure along both sides of the parenchymal isthmus representing an ‘accessory isthmic lobe’ or ‘accessory midline lung’.^8^ Tosun et al. proposed the term congenital pseudo-HL for cases with a horseshoe configuration of lung and an intervening fissure on imaging.^2^ We also concur with the use of the term pseudo-HL on imaging for such cases of HL with an isthmic fissure.

**FIGURE 9:**
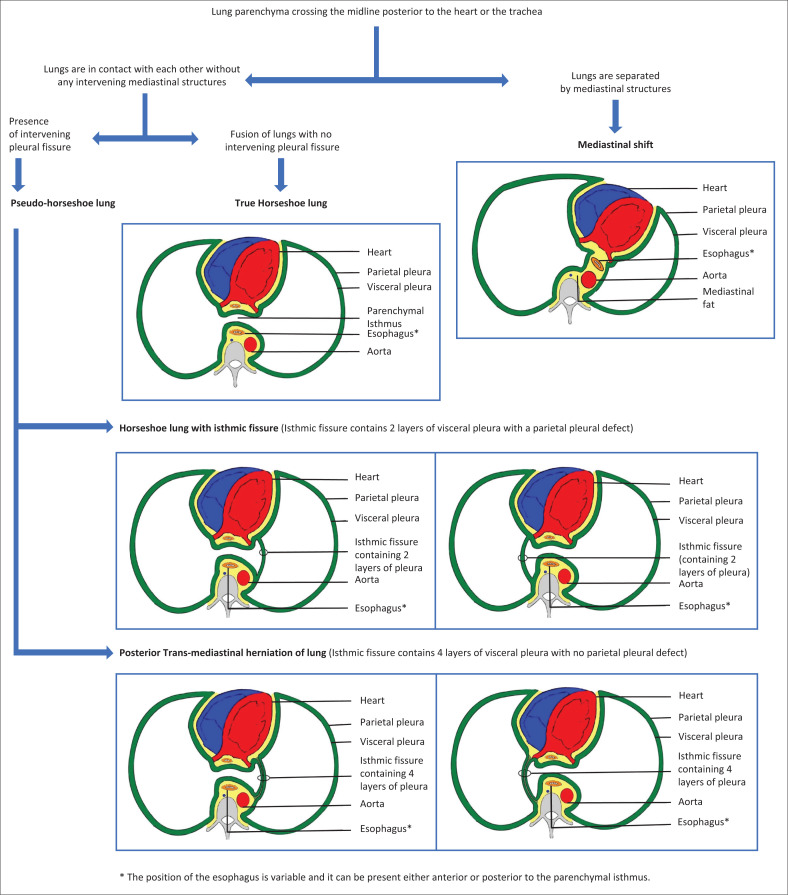
Diagrammatic representation of horseshoe lung, pseudo-horseshoe lung, and posterior trans-mediastinal lung herniation.

In rare instances, trans-mediastinal lung herniation can occur posterior to the heart, and may appear very similar to cases of HL with an isthmic fissure on imaging. In posterior trans-mediastinal lung herniation, the herniated segment of the lung closely abuts the contralateral lung posterior to the heart, forming a horseshoe configuration on imaging. A pleural fissure is visible on CT at the interface of the herniated lung segment with the contralateral lung. However, as cases of trans-mediastinal lung herniation do not have any parietal pleural defect, the intervening fissure comprises both parietal and visceral pleural layers with a total of four pleural layers.^5,6^ Unfortunately, this verification of the number of intervening pleural layers is not possible on imaging and can only be assessed during surgery or at autopsy. As surgery is not always required in cases of HL, we propose that the term pseudo-HL be used on imaging to include both types of cases: HL with isthmic fissure, as well as posterior trans-mediastinal herniation of the lung. This terminology enables differentiation from true HL, in which there is direct fusion of both lungs posterior to the heart, without any intervening fissure (Figure 9).

Fujisawa et al.^5^ reported a case of pulmonary sequestration associated with a horseshoe configuration of the lung, with an intervening fissure on imaging. This case showed systemic arterial supply to the sequestered lung from the descending aorta projecting only onto the right side and venous drainage from the sequestered lung also solely into the right inferior pulmonary vein. Subsequent surgery and histopathology proved a posterior mediastinal herniation of the lung, with no communication between the pleural cavities on either side. Case 4 in this report was also a case of bilateral lower lobe sequestration with HL. Although we lack surgical correlation for this case, the CT showed venous drainage into bilateral inferior pulmonary veins, unlike the case reported by Fujisawa et al. This imaging finding supports the diagnosis of a HL as more likely than trans-mediastinal lung herniation.

Freathy et al.^9^ also reported a case of acquired trans-mediastinal herniation of lung posterior to the heart, after post-operative repair of a thoracoabdominal aortic aneurysm in a patient with Marfan’s disease. However, this condition is extremely uncommon. Manner et al. used the term inverted HL in a foetus showing fusion of the right and left lung apices posterior to the trachea and the oesophagus by a midline isthmus covered by visceral pleura.^10^ In case 1, the parenchymal isthmus extended cranially to the level of tracheal bifurcation and was present posterior to the oesophagus. This case probably represents a bridging case between classical HL and inverted HL, and supports the existence of such cases as a spectrum of the same underlying abnormality. In the second and third cases, the parenchymal isthmus was limited to the basal lung segments.

In the presented cases, the position of the oesophagus with respect to the isthmus of the HL was variable. In cases 1 and 4, the oesophagus was present anterior to the parenchymal isthmus, and in cases 2 and 3 (both cases of pseudo-HL), it was present posterior to the parenchymal isthmus. However, the location of the oesophagus in relation to the isthmus does not help in differentiating true HL from pseudo- horseshoe lung. Location of the oesophagus posterior to the parenchymal isthmus in true HL and anterior to the parenchymal isthmus in pseudo-HL (the opposite of the presented cases in this report) has also been previously documented in the literature.^3,5^

The authors of this article would like to propose that the term ‘pseudo-HL’ should not be used for anterior trans-mediastinal herniation of the lung in the retrosternal region as used by Singh et al. and Siddiqui et al.,^11,12^ in view of the striking differences in their pathogenesis and prognosis when compared with cases of HL with an isthmic fissure. Anterior trans-mediastinal herniation of the lung in the retrosternal region usually occurs secondary to volume loss or destruction of the lung parenchyma and compensatory hyperinflation of the contralateral lung.

The clinical features of cases with HL mainly depend on the associated congenital malformations. Patients with associated cardiovascular malformations and significant left to right shunts in scimitar syndrome usually present early in infancy with features of respiratory distress or pulmonary arterial hypertension, as seen in case 2, where the child was symptomatic since birth. Patients with associated bronchopulmonary malformations usually present with chest infections (typically recurrent), as seen in cases 3 and 4.^7^ Air trapping and bronchial stenosis is common in the isthmic segment of the HL and predisposes the patient to recurrent pulmonary infections.

Horseshoe lung may be associated with various malformations. The most common association is with unilateral lung hypoplasia, invariably involving the right lung; the association of HL with left lung hypoplasia is very rare.^3,13^ This association with left lung hypoplasia was seen in cases 1 and 2. The isthmic lung segment is always supplied by the arterial and bronchial branches of the hypoplastic lung.^1,14^

In up to 80% of cases, HL is associated with scimitar syndrome.^1,15,16^ Scimitar syndrome is characterised by a hypogenetic or hypoplastic lung with partial anomalous pulmonary venous drainage. It typically involves the right lung, with the anomalous draining vein traversing parallel to the right heart border, thus resembling a scimitar (Turkish sword) on the chest radiograph.^15^ Scimitar syndrome is also associated with hypoplasia of the pulmonary artery, and often shows anomalous systemic arterial supply to a part of the hypoplastic lung. Only the cases of HL involving right lung hypoplasia are associated with scimitar syndrome, and no case of HL involving left lung hypoplasia has been reported with scimitar syndrome.^3^ Association of HL with bilateral scimitar syndrome variants as seen in case 2 is extremely rare and only a few cases have been reported in the literature.^14^

Case 3 represented a case of a meandering right pulmonary vein and/or pseudo-scimitar syndrome, in which an anomalous unilateral single pulmonary vein was seen in a hypoplastic right lung, draining normally into the left atrium. Along with HL, this case also had other features of classic scimitar syndrome like hypoplastic right lung and aberrant systemic arterial supply to the right lower lobe, which reinforces the view of previous authors that classic scimitar syndrome and meandering right pulmonary vein or pseudo-scimitar syndrome are closely related pulmonary anomalies having a common embryological basis.^17,18^

Horseshoe lung may be associated with foregut and bronchopulmonary malformations, like abnormalities of the bronchial tree, bilateral intralobar pulmonary sequestration, congenital pulmonary airway malformation, oesophageal atresia with tracheoesophageal fistula and oesophagobronchial fistula.^1,16^ The fourth case was a case of bilateral intralobar pulmonary sequestration associated with HL. Pulmonary sequestration is a developmental anomaly of the lung in which a segment of dysplastic lung tissue is supplied by an anomalous systemic artery and is not connected with the normal tracheobronchial tree.^19^ Intralobar pulmonary sequestration is embedded within the normal lung parenchyma and does not have a separate pleural covering.^20^ To the best of our knowledge, only two cases of bilateral intralobar pulmonary sequestration associated with a bridging isthmus or HL have been described in the literature,^19,20^ apart from the surgically proven case of sequestration with mediastinal lung herniation previously mentioned.^5^

## Conclusion

Horseshoe lung describes the union or abutment of both lungs posterior to the heart, and is characterised by a parietal pleural defect. Some cases may show an intervening isthmic fissure comprising two layers of visceral pleura. The terminology of true HL and pseudo-HL should be used on imaging, the latter encompasses cases of HL with an isthmic fissure, as well as the rare cases of posterior trans-mediastinal lung herniation.

The clinical presentation and prognosis of patients with HL is primarily determined by the nature and severity of associated bronchopulmonary and cardiovascular malformations. The most common association is with unilateral (usually right) lung hypoplasia and scimitar syndrome. Contrast enhanced CT is the imaging modality of choice for accurate diagnosis of these complex anomalies. It is important to follow a systematic approach while describing these lesions and actively look for possible involvement of all components, including lung parenchyma, airway, arterial supply and venous drainage, as well as cardiovascular and other associated anomalies.
